# Mixed crystal of bis­(ammonium/oxonium) tetra­aqua-μ_3_-fluorido-dodeca­kis­(μ_2_-tri­fluoro­acetato)*octa­hedro*-hexa­ytterbiate(III) tetra­hydrate, [(NH_4_)_1–*x*_(H_3_O)_*x*_]_2_[Yb_6_F_8_(O_2_CCF_3_)_12_(H_2_O)_4_]·4H_2_O (*x* = 1/4), containing a hexa­nuclear ytterbium(III) carboxyl­ate complex with face-capping fluoride ligands and comprising an unusual kind of substitutional disorder

**DOI:** 10.1107/S2056989022004790

**Published:** 2022-05-17

**Authors:** Florian Morsbach, Walter Frank

**Affiliations:** a Institut für Anorganische Chemie und Strukturchemie, Lehrstuhl II: Material-, und Strukturforschung, Heinrich-Heine-Universität Düsseldorf, D-40225, Düsseldorf, Germany; Vienna University of Technology, Austria

**Keywords:** crystal structure, ytterbium, perfluoro­carboxyl­ates, *octa­hedro*-hexa­nuclear complex, face-capping fluorido ligands, mixed crystal

## Abstract

The reaction of ytterbium metal with ammonium tri­fluoro­acetate in liquid ammonia resulted in a green substance comprising a substantial amount of ytterbium(II) tri­fluoro­acetate that is a useful precursor for the oxidative synthesis of the new ytterbium(III) compound, [(NH_4_)_1–*x*_(H_3_O)_*x*_]_2_[Yb_6_F_8_(O_2_CCF_3_)_12_(H_2_O)_4_]·4H_2_O (*x* = 1/4), in aqueous tri­fluoro­acetic acid. This mixed ammonium/oxonium crystalline solid is the first example of a substance containing an *octa­hedro*-hexa­nuclear ytterbium(III) complex with μ_3_-face-capping fluorido ligands. The main structural features of its [Yb_6_F_8_] core are non-bonding Yb⋯Yb distances and Yb—F bond lengths of 3.7576 (3)–3.9413 (5) and 2.2375 (17)–2.3509 (17) Å, respectively. Yb—O bond lengths involving the O atoms of *O*,*O*′-bridging carboxyl­ato ligands and vertex-substituting aqua ligands are in the ranges 2.23 (4)–2.329 (2) and 2.448 (2)–2.544 (3) Å, respectively. These bond lengths are in accordance with expectations, taking into account lanthanoid contraction. Inter­estingly, there is a significant ammonium *versus* oxonium ion site dependence, not only of the hydrate water mol­ecule positions within the solid’s hydrogen-bonding framework, but also of the coordination sites of one carboxyl­ato and one aqua ligand in the hexa­nuclear complex.

## Abstract 2

By an oxidative synthesis in aqueous tri­fluoro­acetic acid, the mixed ammonium/oxonium crystalline solid [(NH_4_)_1–*x*_(H_3_O)_*x*_]_2_[Yb_6_F_8_(O_2_CCF_3_)_12_(H_2_O)_4_]·4H_2_O is obtained from ytterbium(II) tri­fluoro­acetate. It is the first example of a substance containing an *octa­hedro*-hexa­nuclear ytterbium(III) complex with μ_3_-face-capping fluorido ligands and comprises an unusual kind of substitutional disorder. The effects of the disordered cation position on the coordination of additional *O*,*O*′-bridging carboxyl­ato and aqua ligands are discussed in detail.

## 1. Chemical context

The stabilizing influence of liquid ammonia as a reaction medium on *Ln* ^II^ of certain lanthanoids (*Ln*) is well known (Warf & Korst, 1956 ▸; Warf, 1970 ▸). Selected ytterbium(II) compounds such as bis­(cyclo­penta­dien­yl)ytterbium(II) (Fischer & Fischer, 1965 ▸; Hayes & Thomas, 1969 ▸), ytterbium(II) phosphide (Pytlewsky & Howell, 1967 ▸), ytterbium(II) amide (Hadenfeldt & Juza, 1969 ▸; Hadenfeldt *et al.*, 1970 ▸; Görne *et al.*, 2016 ▸) and ytterbium(II) halides (Howell & Pytlewski, 1969 ▸) can be obtained by precipitation reactions in liquid ammonia. Adapting this procedure in explorative attempts to synthesize ytterbium(II) tri­fluoro­acetate, we obtained a green mixture of substances, the color of which indicating the presence of Yb^II^ ions. By dissolution experiments in tri­fluoro­acetic acid and subsequent crystallization under non-inert conditions, we obtained colorless crystals of the title compound. The formation of this substance requires not only redox reactions with the change of the oxidation state from 0 to +II and from +II to +III, but also an activation of the C—F bonds of the tri­fluoro­acetate anion (Rillings & Roberts, 1974 ▸). This is evident not only from the presence of fluorido ligands as part of the *octa­hedro*-hexa­nuclear complex anion of the title compound, [(NH_4_)_1–*x*_(H_3_O)_*x*_]_2_[Yb_6_F_8_(O_2_CCF_3_)_12_(H_2_O)_4_]·4H_2_O (*x* = 0.25), but also from the presence of ammonium fluoride in the greenish precipitate from the reaction of ytterbium metal with ammonium tri­fluoro­acetate in liquid ammonia. 
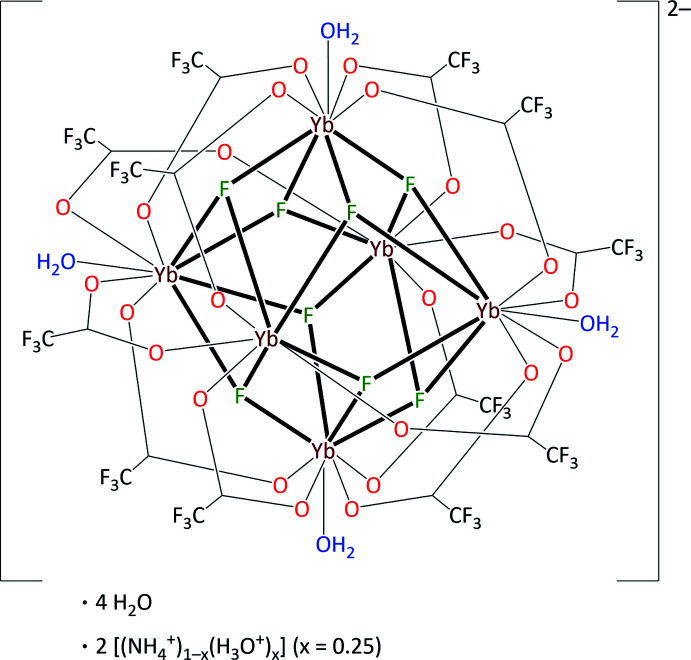


## 2. Structural commentary

**Figure 1:**
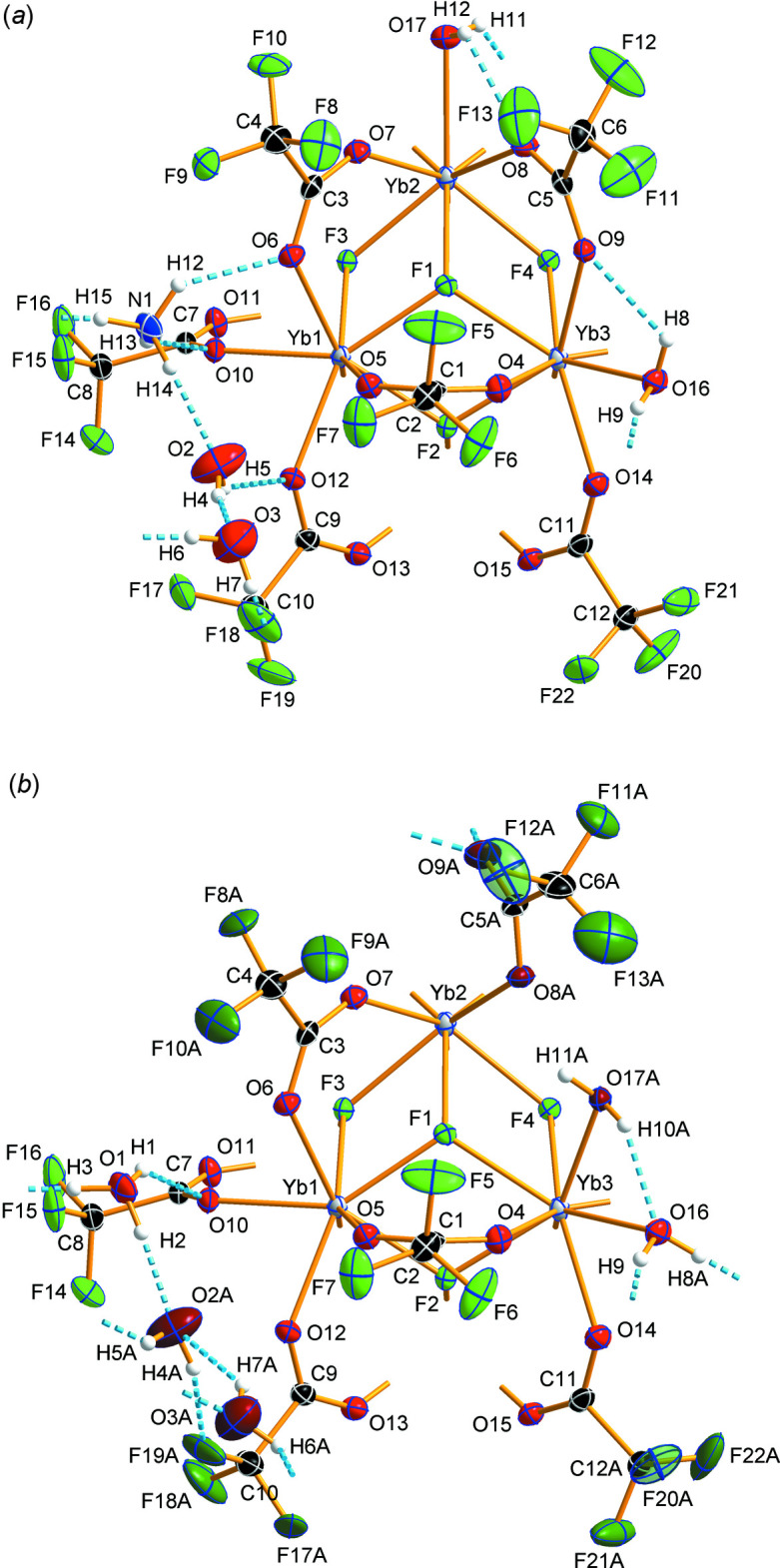
Asymmetric unit of [(NH_4_)_1–*x*_(H_3_O)_*x*_]_2_[Yb_6_F_8_(O_2_CCF_3_)_12_(H_2_O)_4_]·4H_2_O (*x* = 1/4), as related to the NH_4_^+^-containing partial occupation site (*a*) and as related to the H_3_O^+^-containing partial occupation site (*b*), shown separately with the same view direction and the same scaling. Displacement ellipsoids are drawn at the 50% probability level, hydrogen atoms are drawn with an arbitrary radius. The CF_3_ groups at C5 and C11 suffer from rotational disorder that is not related to the cation substitution; only F atoms of the major occupied sites are shown. The directions of further Yb—O and Yb—F bonds are given by truncated sticks, the directions of hydrogen-bonding by segmented blue sticks. Note the coincidence of most parts of the partial occupation site models and the significant differences in the cation/water region and the coordination spheres of Yb2 and Yb3.

**Figure 2:**
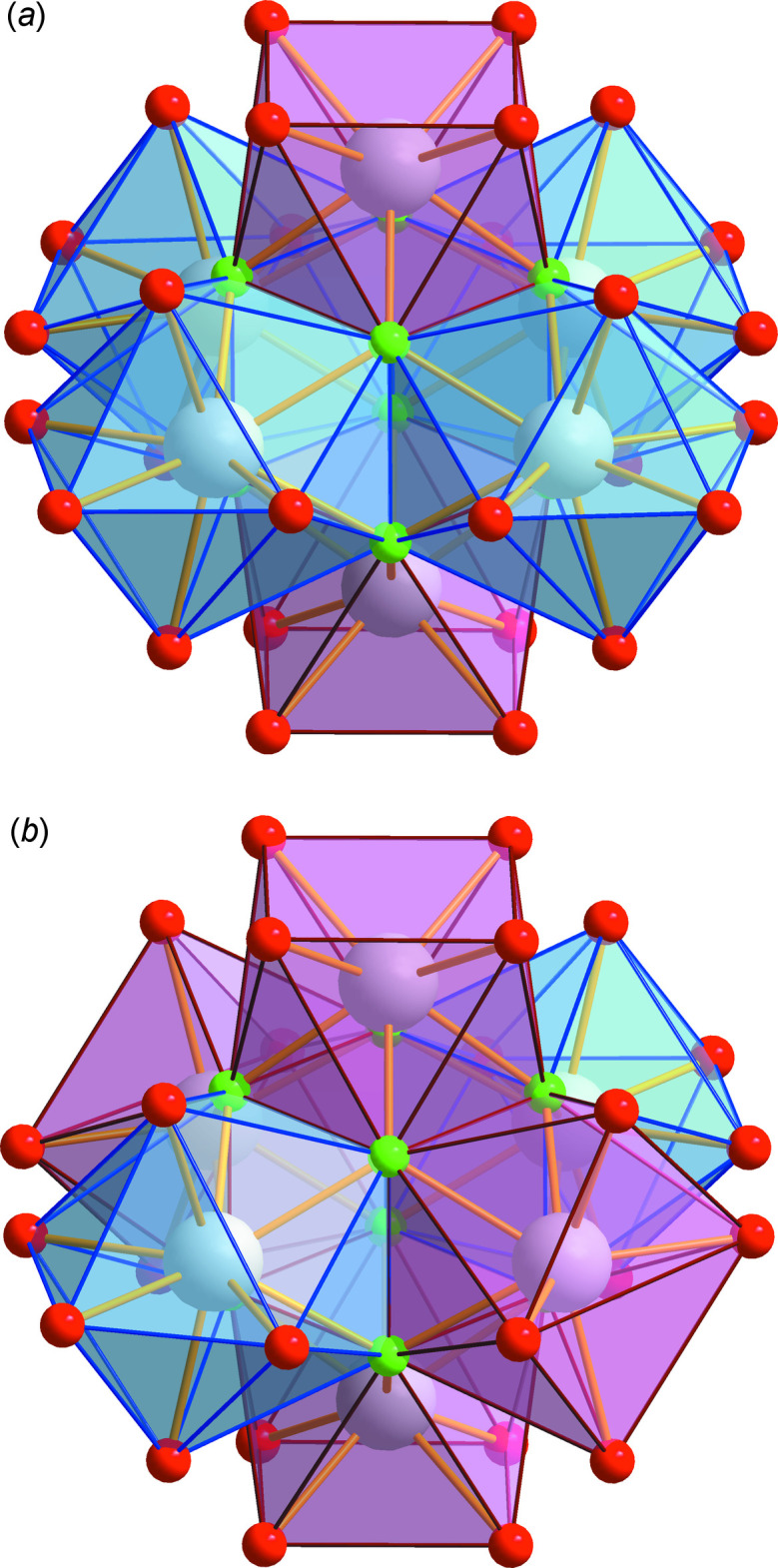
Central [Yb_6_F_8_] core of the title structure with additional O atoms coordinating to Yb^III^ ions, with the cation partial occupation site occupied by NH_4_^+^ (*a*) and H_3_O^+^ (*b*). For Yb^III^ ions with square-anti­prismatic coordination, polyhedra are drawn in red, for Yb^III^ ions with monocapped square-anti­prismatic coordination, polyhedra are drawn in blue; color code: O (red), F (green), Yb (gray).

In the course of the crystal-structure refinement, the crystal under investigation turned out to be a mixed crystal characterized by NH_4_^+^/H_3_O^+^ substitution. However, the structure model with disorder of the cation sites is much more complicated because the disorder not only affects the latter, but also other parts of the crystal structure. Fig. 1 ▸ shows the asymmetric unit of the title compound, separated in terms of the NH_4_^+^-containing partial occupation site (part *a*) and in terms of the H_3_O^+^-containing partial occupation site (part *b*). Both partial occupation site units comprise three Yb^II^ ions, four fluoride anions, six tri­fluoro­acetate anions and two water mol­ecules, all in general position and establishing one half of a centrosymmetric *octa­hedro*-hexa­nuclear [Yb_6_F_8_(O_2_CCF_3_)_12_(H_2_O)_4_]^2–^ complex. Also in general positions, one NH_4_^+^ or H_3_O^+^ cation and two water mol­ecules complete the asymmetric unit. The charge balance of the double-negatively charged complex ion is ensured by two symmetry-related cations. The most prominent moiety in both cases is the *octa­hedro*-hexa­nuclear anionic complex, formed by six Yb^III^ ions with non-bonding Yb⋯Yb distances of 3.7576 (3)–3.9413 (5) Å (mean 3.83 Å, see Table 1 ▸), the eight octa­hedral faces of which are capped by μ_3_-fluorido ligands. In the NH_4_^+^ case, all twelve octa­hedral edges of the central [Yb_6_F_8_] core are bridged by μ_2_-tri­fluoro­acetato ligands. Yb1 is eightfold coordinated with a typical square-anti­prismatic coordination polyhedron (Karraker, 1970 ▸). Water mol­ecules additionally coordinate the octa­hedral vertices of the Yb2 and Yb3 sites and complete the coordination sphere of these Yb^III^ ions, giving a ninefold coordination that results in monocapped square-anti­prismatic coordination polyhedra (Fig. 2 ▸ *a*). In the H_3_O^+^ case, one tri­fluoro­acetato ligand binds to Yb2 monodentately only, while two water mol­ecules coordinate to Yb3 in return (Fig. 1 ▸), giving an eightfold coordination of Yb1 and Yb2 and a ninefold coordination for Yb3 (Fig. 2 ▸ *b*). At first view, the nature of the cation seems to influence the remaining parts of the structure and even to some extent the ligand substitution pattern of the hexa­nuclear complex. However, we cannot exclude the possibility that the presence of the two isomeric anions (related to hydration) is the origin of the cation substitution. The Yb—O bond lengths of 2.23 (4)–2.329 (2) Å (mean 2.30 Å), and the O—C—O′ bond angles of 129.6 (3)–132.2 (3)° (mean 129.9°) of the tri­fluoro­acetato ligands are in typical ranges for the bidentately bridging coordination mode of carboxyl­ate ligands (Rohde & Urland, 2006 ▸). Relevant Yb—F and Yb—O bond lengths are given in Table 1 ▸, along with the corresponding empirical bond valences for each bond, *s*_i_. The Yb—F bond lengths and the bond-valence sums *S* of 3.01–3.13 valence units give striking structural evidence for the presence of fluorido ligands. Comparisons of the complex anion with the one in the very recent crystal-structure determination of an *octa­hedro*-hexa­nuclear terbium(III) complex containing a [Tb_6_F_8_] core (Ling *et al.*, 2020 ▸) and with some europium(III) complexes containing [Eu_6_F_8_] cores (Morsbach *et al.*, 2022 ▸) reveal that the non-bonding *Ln*⋯*Ln* distances [mean 3.97 Å (*Ln* = Tb) and 4.00 Å (*Ln* = Eu)] as well as the *Ln*—F (mean 2.38 and 2.38 Å) and *Ln*—O bond lengths (mean 2.34 and 2.40 Å) in complexes of this type are influenced by the lanthanoid contraction, with these structural parameters decreasing from Eu to Yb due to the smaller ionic radius of Yb^III^ compared to Tb^III^ and Eu^III^: 1.04 Å *vs.* 1.10 Å and 1.12 Å (all values for CN 9; Shannon, 1976 ▸).

## 3. Supra­molecular features

**Figure 3:**
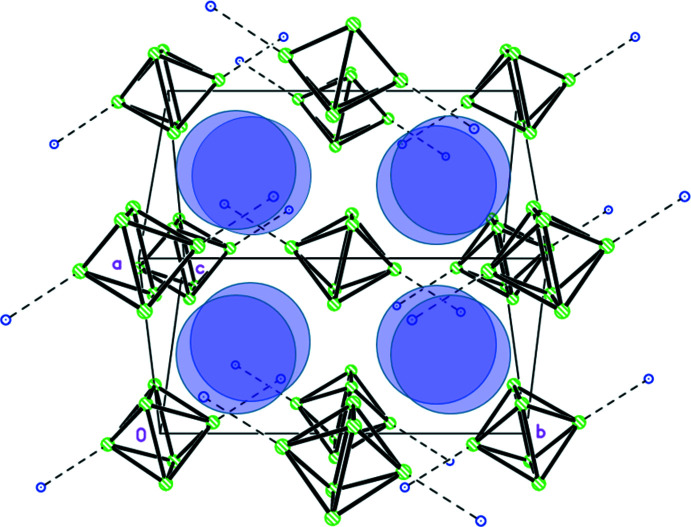
Schematic packing diagram. The bulky fluorido­carboxyl­ate anions are represented by octa­hedra, the cation positions are given by dot-centered circles, and the closest anion⋯cation contacts are indicated by dashed lines. The distorted *fcc*-packing of the bulky anions can easily be recognized. Note the offset of the cations from the centers of the tetra­hedral inter­sticial regions that are indicated by eight translucent circular areas. With respect to the primitive unit cell, this offset is along [101] or in the reverse direction and to a lesser extent along [010] or in the reverse direction. Thus, each cation is significantly closer to one of the four anions establishing a tetra­hedral hole than to the other three.

**Figure 4:**
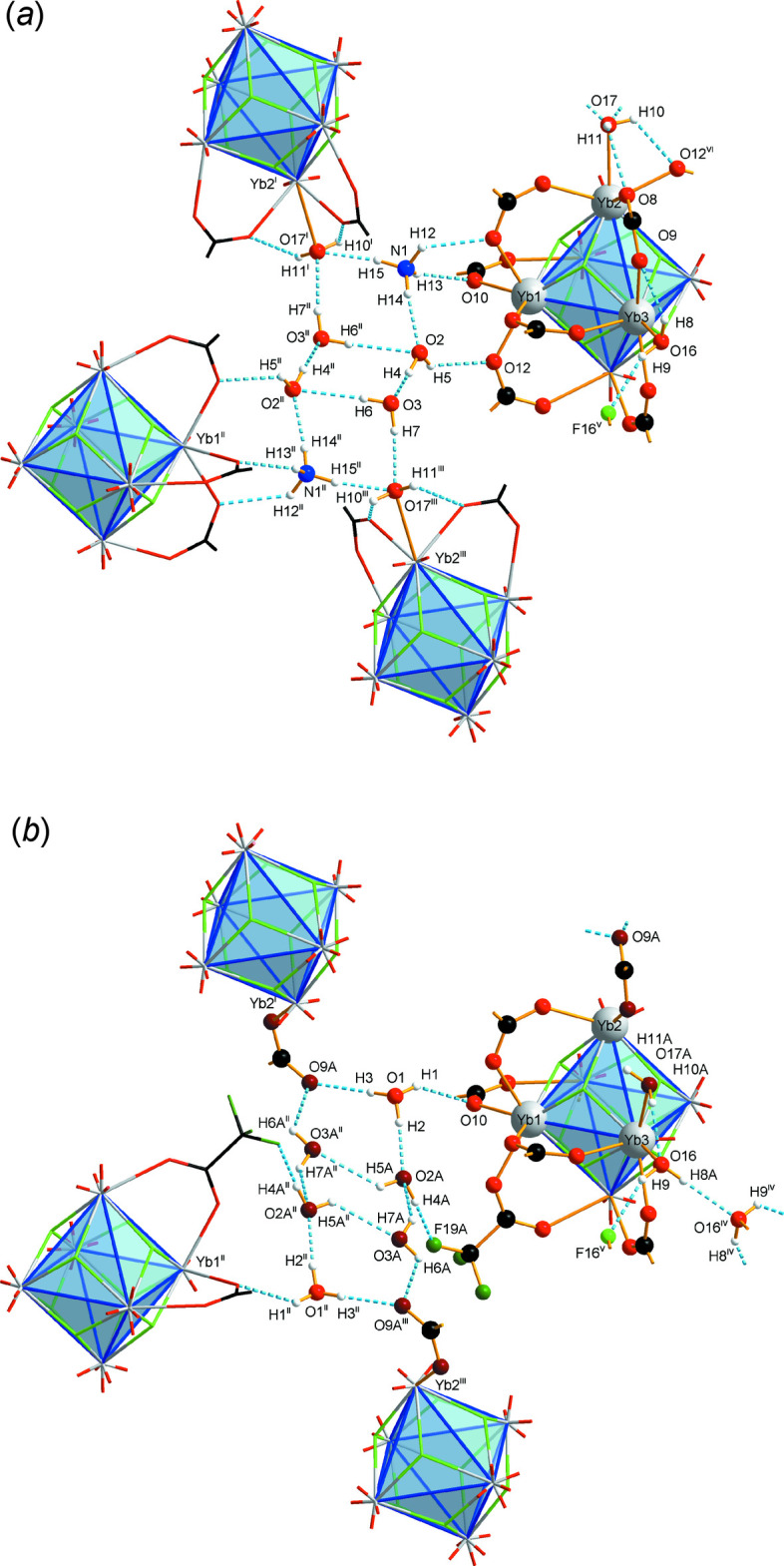
Sections of the extended hydrogen-bonded supra­molecular network of the title compound, with the cation partial occupation site occupied by NH_4_^+^ (*a*) and H_3_O^+^ (*b*). For the sake of clarity, only Yb1, Yb2, Yb3, some symmetry-related Yb atoms, and F, O and H atoms involved in hydrogen bonds are labeled. [Symmetry codes: (i) −*x* + 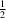 , *y* + 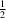 , −*z* + 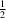 ; (ii) −*x* + 1, −*y* + 2, −*z* + 1; (iii) *x* + 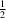 , −*y* + 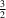 , *z* + 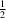 ; (iv) −*x* + 2, −*y* + 1, −*z* + 1; (v) *x* + 1, *y*, *z*; (vi) −*x* + 1, −*y* + 1, −*z* + 1].

Approximating the hexa­nuclear anionic complex as a bulky sphere, a distorted *fcc* packing of these voluminous anions can be recognized. As shown in Fig. 3 ▸ in more detail, in a strongly off-center mode the small cations occupy all tetra­hedral inter­stices of this packing. The hexa­nuclear ytterbiate(III) anions as well as all other moieties are engaged in an extended hydrogen-bonded supra­molecular network (Table 2 ▸). All hydrogen bonds have medium to weak strengths. A remarkable segment of this network is established by two symmetry-related pairs of water mol­ecules around a center of inversion. Depending on the nature of the cation, the positions and orientations of these water mol­ecules are significantly different, as shown in Fig. 4 ▸. Note, that the partial occupation sites occupied by O2 and O3 are related to NH_4_^+^ and those occupied by O2*A* and O3*A* are related to H_3_O^+^. In both cases, the graph set descriptor 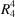 (8) can be assigned to the hydrogen-bond motif (Etter *et al.*, 1990 ▸). However, a different orientation of the hydrogen-bond-donor direction is given within the ring-shaped system. In the NH_4_^+^ case, with the exception of four H atoms at the vertices (H5, H7, H5^ii^ and H7^ii^), the (H_2_O)_4_ unit is almost planar (Fig. 4 ▸ *a*), while in the H_3_O^+^ case, all H atoms are out-of-plane with the O atoms (Fig. 4 ▸ *b*). In both cases, two further four-membered ring motifs are annealed to the (H_2_O)_4_ unit, assigned to the graph-set descriptor 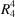 (8). In these motifs, two water mol­ecules, a cation and, in the case of NH_4_^+^ occupying the cation position, an aqua ligand (including O17) from the [Yb_6_F_8_(O_2_CCF_3_)_12_(H_2_O)_4_]^2–^ complex anion are involved. In the case of H_3_O^+^ occupying the cation position, O9*A* from the monodentately bonding tri­fluoro­acetato ligand at Yb2 takes the role of O17 as a double acceptor. With further O—H⋯O′, O—H⋯F, and N—H⋯O hydrogen bonds, the entire tricyclic hydrogen-bonding motif connects in total four of the hexa­nuclear complexes, each of which gives further connections in three symmetry-related directions. As expected, due to the higher solvation free energy of H_3_O^+^ compared to NH_4_^+^ (Taft *et al.*, 1978 ▸; Saielli, 2010 ▸), the primary hydrogen-bonding inter­action of H_3_O^+^ is significantly stronger than that of NH_4_^+^ [O1⋯O2*A* = 2.619 (18) Å *vs*. N1⋯O17^i^ = 2.766 (7) Å].

## 4. Database survey

A search of the Cambridge Structural Database (CSD; version 5.43, update of November 2021; Groom *et al.*, 2016 ▸) resulted in 80 hits for isolated *octa­hedro*-hexa­nuclear lanthanoid complexes with eight μ_3_-face-capping ligands of any type, excluding a μ_6_-central atom. Only two of these contain eight μ_3_-halogenido ligands of any type, including the carboxyl­ato fluorido complex with a [Tb_6_F_8_] core (KUWMOH, Ling *et al.*, 2020 ▸) and a cyclo­penta­dienyl iodido complex with a [Yb_6_I_8_] core (TUFWEW, Constantine *et al.*, 1996 ▸). Six of the 80 complexes are ytterbium complexes, *viz*. the aforementioned iodido complex, three octa-μ_3_-hydroxido complexes (MINVAI, da Cunha *et al.*, 2013 ▸; HELNAQ, Zhang *et al.*, 2018 ▸; XUKCAK, Luo *et al.*, 2020 ▸) and one tetra-μ_3_-oxido­tetra-μ_3_-hydroxido complex (YINFEJ, Feng *et al.*, 2019 ▸). The first, the second and the fourth of these are parts of metal–organic frameworks (MOFs). Furthermore, there is a hexa-μ_3_-oxidodi-μ_3_-hydroxido complex (KIFVAZ, Duan *et al.*, 2018 ▸). A search in the ICSD (version 2021.2; Belsky *et al.*, 2002 ▸) for structures containing both NH_4_^+^ and H_3_O^+^ ions, resulted in ten hits. Seven of these show NH_4_^+^/H_3_O^+^ substitutional disorder. Three of the seven disordered structures are mixed ammonio­jarosite–hydro­niumjarosite phases, (NH_4_)_1–*x*_(H_3_O)_*x*_Fe_3_(SO_4_)_2_(OH)_6_ (#16020–16022, Basciano & Peterson, 2007 ▸). Furthermore, there are two phosphates (#73847–73848, Ferey *et al.*, 1993 ▸), a molybdatophosphate (#212, Boeyens *et al.*, 1976 ▸) and an oxide (#37066, Thomas & Farrington, 1983 ▸). However, for none of these structures cation-dependent further partial occupation sites are reported.

## 5. Synthesis and crystallization

All chemicals were obtained from commercial sources and used as purchased. In a representative experiment, 0.584 g (0.337 mmol) of ytterbium were dissolved in approximately 50 ml of liquid ammonia (dried over sodium) to which 0.903 g (0.675 mmol) of ammonium tri­fluoro­acetate were added. The ammonia was evaporated, and the residue was dried *in vacuo* until a pressure of 10^−3^ hPa was reached. 0.816 g of a greenish powder were obtained. 100 mg of this powder were stirred in 2 ml of anhydrous tri­fluoro­acetic acid, and the insoluble portions were allowed to settle overnight. The supernatant solution was transferred into an ampoule and stored open in air. Colorless crystals of the title compound grew within one week. A suitable single crystal for X-ray crystal structure determination was selected directly from the mother liquor. An IR spectrum was recorded with a *Spectrum Two* FT–IR spectrometer (Perkin Elmer Inc., 2008 ▸), equipped with a LiTaO_3_ detector (4000–350 cm^−1^) and an ATR unit. Band assignments were made according to metal tri­fluoro­acetate salts (Baillie *et al.*, 1968 ▸; Faniran & Patel, 1976 ▸): *ν*(O—H): 3374, 3287 (*w*); *ν*_as_(COO): 1665 (*s*); 1613 (*m*); 1569 (*m*); *ν*_s_(COO): 1473 (*m*); 1342 (*w*); *ν*(C—F): 1204, 1142 (*s*); *ν*(C–C): 849 (*m*); *δ*(CF_3_): 798 (*m*); *δ*(O—C—O): 724 (*s*); 687 (*w*); *δ*(CF_3_): 613, 522, 452 (*vw*). A CHN analysis was performed with a *vario MICRO cube* (Elementar Analysensysteme GmbH, 2015 ▸). Analysis calculated for C_24_H_23.50_N_1.50_O_32.50_F_44_Yb_6_ (2727.15 g mol^−1^): C 10.57, H 0.87, N 0.77; found: C 10.7, H 0.8, N 1.0.

## 6. Refinement

Crystal data along with data collection and structure refinement details are summarized in Table 3 ▸. After having completed the primary structural model, (*a*) physically non-meaningful anisotropic displacement parameters, (*b*) features appearing in the difference-electron density map in the course of further refinement cycles and (*c*) analysis of potential hydrogen-bonding orientations clearly indicated disorder that refers to: (i) position and nature of the cation (NH_4_^+^ *vs.* H_3_O^+^), (ii) position and coordination mode of the complete carboxyl­ato ligand with atoms O8 and O9, (iii) position (coordination site) of the aqua ligand with O17, (iv) orientation of the aqua ligand with O16, (v) rotational orientation of four of the six CF_3_ groups and (vi) position and orientation of the two hydrate water mol­ecules. The refinement of site-occupation factors finally proved the disorder according to (i), (ii), (iii), (iv), (vi) and the rotational orientations of three of the four CF_3_ groups addressed in (v) to be directly dependent. In the final stages of a converging refinement, for these dependent sites a common occupation factor was introduced and refined to 0.749 (4) for NH_4_^+^ and its related partial occupation site moieties, giving 0.251 (4) for H_3_O^+^ and its related moieties. When involved in disorder, NH_4_^+^ and H_3_O^+^ ions can hardly be distinguished in a structure refinement based on X-ray diffraction data alone. All substances related to the class of the title compound showed somewhat too high proportions for N in the combustion analysis, and due to the complex vibration spectra, an identification of O—H or N—H stretching modes in the IR spectrum is not possible. In consequence, the nature of the cations could not be determined by chemical analysis or spectroscopic studies. Even though the crystal structure model is therefore based only on the results of structure refinement and comparative structural considerations, the final choice of occupation with NH_4_^+^ and H_3_O^+^ is unambiguous for the following reasons: the partial occupation site related to N1 with 75% occupation shows four tetra­hedrally arranged residual electron-density maxima, which are identified as H atoms on the basis of their heights and spacings; at the site related to O1 with 25% occupation, clear electron-density maxima could not be identified, as expected. However, comparative refinements of the occupation factors showed in case of occupation of both partial occupation sites with O atoms clearly too small [Σs.o.f.(O,O) = 0.88 (3)], in case of occupation of both partial sites with N atoms a clearly too large value [Σs.o.f.(N,N) = 1.13 (3)] of the sum of the occupation factors. In the case of the occupation of the higher-populated site with N and the lower-populated site with O, a value close to one [Σs.o.f.(N,O) = 1.03 (3)] resulted. Within the network of hydrogen bonds, the N⋯O distance of the shortest N—H⋯O bond [2.766 (7) Å] fits well to the expectations taking into account the optimized calculated shape of the hydrated ammonium ion [NH_4_^+^—OH_2_ = 2.728 Å, NH_4_^+^—(OH_2_)_2_ = 2.784, 2.785 Å, NH_4_^+^—(OH_2_)_3_ = 2.832 Å (3×), at the B3LYP/6-31*G* level of theory; Jiang *et al.*, 1999 ▸]. The much shorter O⋯O distance of 2.619 (18) Å from the lower-occupied site to the O atom of the next water mol­ecule is typical for comparatively strong O—H⋯O hydrogen bonds, but out of the limits of expectation for N—H⋯O bonds to water mol­ecules [Meot-Ner (Mautner), 2005 ▸]. Finally, if the lower-occupied site were assumed to be a NH_4_^+^ ion, no suitable hydrogen-bond acceptor could be identified for an additional, fourth hydrogen bond. All disordered parts of the structure were subjected to appropriate bond lengths and angles and anisotropic displacement restraints or constraints. The C—F bond lengths of the disordered CF_3_ groups related to C4, C10, C12, (C6) were restrained to 1.32 Å within a s. u. of 0.02 Å (0.002 Å), combined with default F⋯F same distance and with strongly restrictive isotropic displacement restraints for all F atoms. No restraints were needed for the two CF_3_ groups not suffering from disorder. For the CF_3_ group related to C6, which suffers from both dependent positional and independent rotational disorder, more restrictive C—F bond lengths restraints (see above) had to be used and the C—C bond length was restrained to 1.52 Å within a s. u. of 0.02 Å. For atoms at partial occupation sites in close proximity, in an approximative manner equivalent anisotropic displacement constraints have been applied, namely for the pairs N1/O1, O2/O2*A*, O3/O3*A*, O8/O8*A*, O9/O17*A*, C12/C12*A*. The NH_4_^+^ ion was treated in the refinement as a rigid group with idealized tetra­hedral shape and N—H bond lengths constrained to 0.91 Å. The H_3_O^+^ cation was included as a rigid flat pyramid with O—H bond lengths constrained to 0.84 Å and the pyramidalization defined by H⋯H distances constrained to 1.39 Å. The hydrate water mol­ecules related to O2 and O2*A* were treated as rigid groups with O—H bond lengths of 0.83 Å and H—O—H angles adjusted to 105.4°. The O—H bond lengths of the aqua ligands including O16, O16*A*, O17, O17*A* and of the hydrate water mol­ecules including O3 and O3*A* were restrained to 0.83 Å within an s.u. of 0.02 Å, the corres­ponding H⋯H distances to 1.32 Å within an s.u. of 0.04 Å defining H—O—H angles of 105 (4)–109 (4)°. *U*_iso_(H) values of all H atoms were set to 1.5*U*_eq_ of the parent atoms.

## Supplementary Material

Crystal structure: contains datablock(s) I. DOI: 10.1107/S2056989022004790/wm5637sup1.cif

Structure factors: contains datablock(s) I. DOI: 10.1107/S2056989022004790/wm5637Isup2.hkl

CCDC reference: 2170493

Additional supporting information:  crystallographic information; 3D view; checkCIF report

## Figures and Tables

**Table 1:** Selected structural parameters (Å) and empirical bond valences *s*_i_ (valence units) for Yb1–Yb3 Calculation of empirical bond valences according to: *S* = Σ *s*_i_ = Σ{exp [(*d* – *d*_0_) / *B*]} (Brown & Altermatt, 1985 ▸), with *d*_0_(Yb^III^—F) = 1.875 Å, *B* = 0.37 (Brese & O’Keeffe, 1991 ▸) and *d*_0_(Yb^III^—O) = 1.965 Å, *B* = 0.37 (Brown & Altermatt, 1985 ▸).

*X*—*Y*	*d*_i_	s.o.f. of atom *Y*	*s*_i_
Yb1—F1	2.2375 (17)	1	0.38
Yb1—F2	2.2382 (17)	1	0.37
Yb1—F3	2.2431 (17)	1	0.37
Yb1—F4^vi^	2.2444 (17)	1	0.37
Yb1—O5	2.273 (2)	1	0.43
Yb1—O10	2.291 (2)	1	0.41
Yb1—O6	2.306 (2)	1	0.40
Yb1—O12	2.309 (2)	1	0.39
			*S* = 3.13
Yb2—F2^vi^	2.2895 (17)	1	0.33
Yb2—F4	2.3035 (17)	1	0.31
Yb2—F3	2.3061 (17)	1	0.31
Yb2—F1	2.3276 (17)	1	0.29
Yb2—O8*A*	2.23 (4)	0.251 (4)	0.12
Yb2—O15^vi^	2.286 (2)	1	0.42
Yb2—O8	2.299 (13)	0.749 (4)	0.30
Yb2—O7	2.303 (2)	1	0.40
Yb2—O13^vi^	2.329 (2)	1	0.37
Yb2—O17	2.544 (3)	0.749 (4)	0.16
			*S* = 3.02
Yb3—F3^vi^	2.3210 (17)	1	0.30
Yb3—F2	2.3316 (17)	1	0.29
Yb3—F1	2.3321 (17)	1	0.29
Yb3—F4	2.3509 (17)	1	0.28
Yb3—O17*A*	2.27 (4)	0.251 (4)	0.11
Yb3—O14	2.290 (2)	1	0.42
Yb3—O9	2.312 (11)	0.749 (4)	0.29
Yb3—O11^vi^	2.321 (2)	1	0.38
Yb3—O4	2.323 (2)	1	0.38
Yb3—O16	2.448 (2)	1	0.27
			*S* = 3.01
			
Yb1⋯Yb2^vi^	3.7576 (3)	Yb2⋯Yb3^vi^	3.9020 (6)
Yb1⋯Yb2	3.7828 (3)	Yb2⋯Yb3	3.9431 (5)
Yb1⋯Yb3	3.8018 (3)	Yb3⋯Yb1	3.8018 (3)
Yb1⋯Yb3^vi^	3.8163 (3)	Yb3⋯Yb1^vi^	3.8163 (3)
Yb2⋯Yb1^vi^	3.7576 (3)	Yb3⋯Yb2^vi^	3.9020 (6)
Yb2⋯Yb1	3.7828 (3)	Yb3⋯Yb2	3.9431 (5)

Symmetry code: (vi) −*x* + 1, −*y* + 1, −*z* + 1.

**Table 2:** Hydrogen-bond geometry (Å, °)

*D*—H⋯*A*	*D*—H	H⋯*A*	*D*⋯*A*	*D*—H⋯*A*
N1—H12⋯O6	0.91	2.21	2.883 (8)	131
N1—H15⋯O17^i^	0.91	1.86	2.766 (7)	172
N1—H13⋯O10	0.91	2.08	2.853 (10)	142
N1—H14⋯O2	0.91	1.95	2.837 (9)	165
O1—H2⋯O2*A*	0.84	1.78	2.619 (18)	175
O1—H3⋯O9*A* ^i^	0.84	2.01	2.842 (16)	173
O1—H1⋯O10	0.84	2.30	3.03 (3)	145
O2—H4⋯O3	0.83 (1)	1.91 (3)	2.704 (11)	159 (7)
O2—H5⋯O12	0.83 (1)	2.31 (5)	2.978 (7)	138 (6)
O3—H6⋯O2^ii^	0.85 (2)	2.22 (2)	3.055 (10)	167 (8)
O3—H7⋯O17^iii^	0.85 (2)	2.01 (2)	2.835 (9)	163 (8)
O2*A*—H4*A*⋯F19*A*	0.83 (1)	1.90 (7)	2.68 (2)	156 (17)
O2*A*—H5*A*⋯O3*A* ^ii^	0.83 (1)	2.35 (14)	2.96 (3)	131 (16)
O3*A*—H7*A*⋯O2*A*	0.84 (2)	2.20 (15)	2.91 (4)	143 (23)
O3*A*—H6*A*⋯O9*A* ^iii^	0.83 (2)	2.6 (2)	3.03 (4)	115 (19)
O16—H8⋯O9	0.83 (2)	2.28 (6)	2.639 (10)	107 (4)
O16—H8*A*⋯O16^iv^	0.84 (2)	2.07 (2)	2.903 (5)	179 (17)
O16—H9⋯F16^v^	0.82 (2)	2.17 (2)	2.954 (3)	160 (4)
O17—H11⋯O8	0.84 (2)	2.25 (8)	2.593 (7)	105 (7)
O17—H10⋯O13^vi^	0.81 (2)	2.22 (5)	2.754 (4)	123 (5)
O17*A*—H10*A*⋯O16	0.83 (1)	1.92 (2)	2.56 (3)	134 (5)

Symmetry codes: (i) 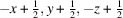 ; (ii) 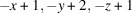 ; (iii) 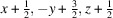 ; (iv) 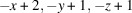 ; (v) 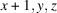 ; (vi) 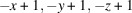 .

**Table 3:** Experimental details

Crystal data	
Chemical formula	[(NH_4_)_1–*x*_(H_3_O)_*x*_]_2_[Yb_6_F_8_(O_2_CCF_3_)_12_(H_2_O)_4_]·4H_2_O (*x* = 1/4)
*M*_r_	2727.15
Crystal system, space group	Monoclinic, *P*2_1_/*n*
Temperature (K)	120
*a*, *b*, *c* (Å)	12.1449 (13), 17.5051 (16), 15.1885 (16)
β (°)	102.999 (4)
*V* (Å^3^)	3146.3 (6)
*Z*	2
Radiation type	Mo *K*α
μ (mm^−1^)	9.04
Crystal size (mm)	0.17 × 0.11 × 0.05

Data collection	
Diffractometer	Bruker APEXII CCD
Absorption correction	Multi-scan (*SADABS*; Krause *et al.*, 2015 ▸)
*T*_min_, *T*_max_	0.665, 1.000
No. of measured, independent and observed [*I* > 2σ(*I*)] reflections	48765, 7213, 6768
*R*_int_	0.035
(sin θ/λ)_max_ (Å^−1^)	0.650

Refinement	
*R*[*F* ^2^ > 2σ(*F* ^2^)], *wR*(*F* ^2^), *S*	0.019, 0.044, 1.08
No. of reflections	7213
No. of parameters	717
No. of restraints	268
H-atom treatment	H atoms treated by a mixture of independent and constrained refinement
Δρ_max_, Δρ_min_ (e Å^−3^)	0.96, −0.89

Computer programs: *APEX2* and *SAINT* (Bruker, 2014 ▸), *SHELXT* (Sheldrick, 2015*a* ▸), *SHELXL* (Sheldrick, 2015*b* ▸), *DIAMOND* (Brandenburg, 2020 ▸), *SHELXTL* (Sheldrick, 2008 ▸) and *publCIF* (Westrip, 2010 ▸).

## supplementary crystallographic information

### Crystal data

**Table:**

(NH_4_)_1.5_(H_3_O)_0.5_[Yb_6_(C_2_F_3_O_2_)_12_F_8_(H_2_O)_4_]·4H_2_O	*F*(000) = 2508
*M**_r_* = 2727.15	*D*_x_ = 2.879 Mg m^−^^3^
Monoclinic, *P*2_1_/*n*	Mo *K*α radiation, λ = 0.71073 Å
*a* = 12.1449 (13) Å	Cell parameters from 9176 reflections
*b* = 17.5051 (16) Å	θ = 2.3–30.6°
*c* = 15.1885 (16) Å	µ = 9.04 mm^−^^1^
β = 102.999 (4)°	*T* = 120 K
*V* = 3146.3 (6) Å^3^	Block, colorless
*Z* = 2	0.17 × 0.11 × 0.05 mm

### Data collection

**Table:**

Bruker APEXII CCD diffractometer	6768 reflections with *I* > 2σ(*I*)
Radiation source: sealed tube	*R*_int_ = 0.035
φ and ω scans	θ_max_ = 27.5°, θ_min_ = 1.8°
Absorption correction: multi-scan (*SADABS*; Krause *et al.*, 2015)	*h* = −15→15
*T*_min_ = 0.665, *T*_max_ = 1.000	*k* = −22→22
48765 measured reflections	*l* = −19→19
7213 independent reflections	

### Refinement

**Table:**

Refinement on *F*^2^	Primary atom site location: structure-invariant direct methods
Least-squares matrix: full	Secondary atom site location: difference Fourier map
*R*[*F*^2^ > 2σ(*F*^2^)] = 0.019	Hydrogen site location: difference Fourier map
*wR*(*F*^2^) = 0.044	H atoms treated by a mixture of independent and constrained refinement
*S* = 1.08	*w* = 1/[σ^2^(*F*_o_^2^) + (0.0153*P*)^2^ + 6.7213*P*] where *P* = (*F*_o_^2^ + 2*F*_c_^2^)/3
7213 reflections	(Δ/σ)_max_ = 0.002
717 parameters	Δρ_max_ = 0.96 e Å^−^^3^
268 restraints	Δρ_min_ = −0.89 e Å^−^^3^

### Special details

**Table:**

Geometry. All esds (except the esd in the dihedral angle between two l.s. planes) are estimated using the full covariance matrix. The cell esds are taken into account individually in the estimation of esds in distances, angles and torsion angles; correlations between esds in cell parameters are only used when they are defined by crystal symmetry. An approximate (isotropic) treatment of cell esds is used for estimating esds involving l.s. planes.

### Fractional atomic coordinates and isotropic or equivalent isotropic displacement parameters (Å^2^)

**Table:**

	*x*	*y*	*z*	*U*_iso_*/*U*_eq_	Occ. (<1)
Yb1	0.45797 (2)	0.64420 (2)	0.47775 (2)	0.01292 (4)	
Yb2	0.41912 (2)	0.47533 (2)	0.31832 (2)	0.01477 (4)	
Yb3	0.71762 (2)	0.52450 (2)	0.47250 (2)	0.01390 (4)	
F1	0.54171 (14)	0.56846 (10)	0.39369 (11)	0.0148 (3)	
F2	0.61494 (14)	0.59078 (9)	0.56047 (12)	0.0150 (3)	
F3	0.34576 (14)	0.54633 (10)	0.41953 (11)	0.0144 (3)	
F4	0.58154 (14)	0.43124 (9)	0.41390 (11)	0.0145 (3)	
F5	0.7250 (3)	0.77519 (17)	0.30917 (17)	0.0626 (9)	
F6	0.85133 (18)	0.78640 (15)	0.43135 (19)	0.0484 (7)	
F7	0.6979 (2)	0.84737 (12)	0.4135 (2)	0.0457 (6)	
F8	0.4230 (4)	0.7285 (3)	0.1531 (4)	0.0512 (14)	0.749 (4)
F9	0.2777 (4)	0.7738 (3)	0.1940 (5)	0.0304 (11)	0.749 (4)
F10	0.2603 (5)	0.6762 (4)	0.1066 (4)	0.0437 (14)	0.749 (4)
F8A	0.2313 (12)	0.6858 (12)	0.1283 (11)	0.048 (5)	0.251 (4)
F9A	0.4019 (14)	0.7031 (10)	0.1268 (11)	0.056 (5)	0.251 (4)
F10A	0.3186 (15)	0.7817 (9)	0.1959 (18)	0.053 (6)	0.251 (4)
F11	0.7963 (4)	0.5569 (5)	0.1769 (4)	0.079 (2)	0.524 (2)
F12	0.6733 (9)	0.4814 (4)	0.1043 (6)	0.077 (3)	0.524 (2)
F13	0.6286 (6)	0.5964 (4)	0.1241 (5)	0.069 (2)	0.524 (2)
O8	0.5527 (8)	0.4900 (7)	0.2337 (7)	0.0203 (13)	0.749 (4)
O9	0.7214 (8)	0.5232 (4)	0.3210 (7)	0.0178 (12)	0.749 (4)
C5	0.6511 (4)	0.5141 (2)	0.2478 (3)	0.0176 (9)	0.749 (4)
C6	0.6887 (3)	0.5381 (2)	0.1630 (3)	0.0293 (12)	0.524 (2)
F11A	0.5725 (10)	0.4478 (6)	0.0149 (6)	0.051 (3)	0.251 (4)
F12A	0.5402 (13)	0.5632 (6)	0.0381 (10)	0.081 (5)	0.251 (4)
F13A	0.6809 (12)	0.5075 (12)	0.1199 (14)	0.082 (6)	0.251 (4)
O8A	0.532 (3)	0.482 (2)	0.222 (3)	0.0203 (13)	0.251 (4)
O9A	0.3984 (10)	0.4521 (6)	0.1033 (8)	0.031 (3)	0.251 (4)
C5A	0.4914 (10)	0.4755 (7)	0.1383 (8)	0.017 (3)	0.251 (4)
C6A	0.5735 (11)	0.4983 (5)	0.0796 (6)	0.036 (4)	0.251 (4)
C6B	0.6887 (3)	0.5381 (2)	0.1630 (3)	0.0293 (12)	0.2247 (11)
F11B	0.6105 (11)	0.5475 (10)	0.0931 (9)	0.059 (4)	0.2247 (11)
F12B	0.7644 (11)	0.4909 (7)	0.1435 (8)	0.046 (3)	0.2247 (11)
F13B	0.7478 (12)	0.6044 (7)	0.1744 (8)	0.047 (3)	0.2247 (11)
F14	0.1535 (2)	0.72832 (17)	0.63021 (17)	0.0524 (7)	
F15	0.1150 (2)	0.78175 (12)	0.50072 (18)	0.0403 (6)	
F16	0.01836 (18)	0.68636 (13)	0.52708 (19)	0.0430 (6)	
F17	0.4294 (4)	0.8118 (3)	0.7341 (4)	0.058 (2)	0.608 (11)
F18	0.5947 (6)	0.8386 (3)	0.7221 (4)	0.053 (2)	0.608 (11)
F19	0.5704 (8)	0.7692 (3)	0.8300 (3)	0.065 (3)	0.608 (11)
F17A	0.6285 (6)	0.7902 (5)	0.8017 (6)	0.045 (3)	0.392 (11)
F18A	0.4547 (8)	0.7724 (5)	0.7927 (7)	0.060 (3)	0.392 (11)
F19A	0.5093 (10)	0.8468 (3)	0.7011 (4)	0.050 (3)	0.392 (11)
C12	0.9654 (5)	0.5666 (4)	0.7508 (4)	0.0251 (11)	0.749 (4)
F20	0.9848 (3)	0.5158 (3)	0.8172 (3)	0.0571 (12)	0.749 (4)
F21	1.0494 (4)	0.5633 (3)	0.7093 (3)	0.0490 (14)	0.749 (4)
F22	0.9698 (3)	0.6338 (2)	0.7904 (3)	0.0528 (12)	0.749 (4)
C12A	0.9711 (13)	0.5553 (11)	0.7414 (13)	0.0251 (11)	0.251 (4)
F20A	1.0361 (15)	0.5984 (8)	0.7057 (12)	0.054 (5)	0.251 (4)
F21A	0.9744 (10)	0.5820 (11)	0.8220 (8)	0.066 (4)	0.251 (4)
F22A	1.0184 (9)	0.4883 (6)	0.7513 (10)	0.061 (4)	0.251 (4)
N1	0.2926 (7)	0.8306 (4)	0.3919 (6)	0.0273 (12)	0.749 (4)
H12	0.301083	0.802939	0.343121	0.041*	0.749 (4)
H13	0.265981	0.799714	0.430668	0.041*	0.749 (4)
H14	0.360537	0.850338	0.420455	0.041*	0.749 (4)
H15	0.242715	0.869256	0.373385	0.041*	0.749 (4)
O1	0.2814 (19)	0.8472 (13)	0.4011 (18)	0.0273 (12)	0.251 (4)
H1	0.264846	0.800676	0.402434	0.041*	0.251 (4)
H2	0.337858	0.858452	0.442138	0.041*	0.251 (4)
H3	0.225795	0.875305	0.402624	0.041*	0.251 (4)
O2	0.5001 (5)	0.8750 (4)	0.5075 (4)	0.0683 (19)	0.749 (4)
H4	0.544 (5)	0.911 (3)	0.525 (4)	0.102*	0.749 (4)
H5	0.496 (7)	0.851 (3)	0.554 (2)	0.102*	0.749 (4)
O3	0.6573 (7)	0.9867 (5)	0.5240 (5)	0.0665 (17)	0.749 (4)
H6	0.619 (6)	1.027 (3)	0.509 (6)	0.100*	0.749 (4)
H7	0.711 (5)	0.998 (4)	0.569 (4)	0.100*	0.749 (4)
O2A	0.4546 (17)	0.8903 (14)	0.5274 (14)	0.0683 (19)	0.251 (4)
H4A	0.491 (13)	0.879 (7)	0.579 (4)	0.102*	0.251 (4)
H5A	0.46 (2)	0.9376 (15)	0.525 (9)	0.102*	0.251 (4)
O3A	0.668 (3)	0.9730 (17)	0.5589 (18)	0.0665 (17)	0.251 (4)
H6A	0.729 (11)	0.956 (12)	0.590 (16)	0.100*	0.251 (4)
H7A	0.628 (16)	0.934 (8)	0.543 (18)	0.100*	0.251 (4)
O4	0.74437 (19)	0.65322 (13)	0.44643 (16)	0.0212 (5)	
O5	0.59075 (19)	0.72520 (12)	0.44714 (15)	0.0194 (5)	
O6	0.38017 (19)	0.69039 (13)	0.33511 (15)	0.0198 (5)	
O7	0.35962 (19)	0.58759 (13)	0.24358 (15)	0.0203 (5)	
O10	0.28607 (18)	0.69187 (12)	0.48928 (15)	0.0183 (4)	
O11	0.18769 (19)	0.59112 (13)	0.52273 (16)	0.0214 (5)	
O12	0.49566 (19)	0.72565 (12)	0.60049 (15)	0.0185 (5)	
O13	0.5663 (2)	0.65211 (13)	0.72162 (15)	0.0208 (5)	
O14	0.84720 (19)	0.54572 (14)	0.60512 (16)	0.0226 (5)	
O15	0.76951 (18)	0.55146 (13)	0.72604 (15)	0.0204 (5)	
O16	0.9075 (2)	0.54443 (14)	0.44699 (17)	0.0219 (5)	
H8	0.906 (5)	0.538 (3)	0.3925 (16)	0.033*	0.749 (4)
H8A	0.960 (8)	0.519 (4)	0.478 (9)	0.033*	0.251 (4)
H9	0.928 (4)	0.5886 (13)	0.458 (3)	0.033*	
O17	0.3486 (3)	0.4558 (2)	0.1490 (2)	0.0263 (8)	0.749 (4)
H10	0.358 (5)	0.4098 (12)	0.151 (4)	0.039*	0.749 (4)
H11	0.396 (6)	0.475 (3)	0.123 (5)	0.039*	0.749 (4)
O17A	0.731 (3)	0.5080 (16)	0.327 (2)	0.0178 (12)	0.251 (4)
H10A	0.796 (3)	0.525 (6)	0.338 (3)	0.027*	0.251 (4)
H11A	0.692 (6)	0.538 (4)	0.290 (6)	0.027*	0.251 (4)
C1	0.6872 (3)	0.71285 (17)	0.4349 (2)	0.0168 (6)	
C2	0.7424 (3)	0.7812 (2)	0.3970 (2)	0.0237 (7)	
C3	0.3584 (3)	0.65671 (18)	0.2611 (2)	0.0170 (6)	
C4	0.3288 (3)	0.7092 (2)	0.1773 (2)	0.0280 (8)	
C7	0.2064 (3)	0.65978 (18)	0.5141 (2)	0.0176 (6)	
C8	0.1216 (3)	0.7149 (2)	0.5428 (2)	0.0241 (7)	
C9	0.5313 (3)	0.71245 (18)	0.6826 (2)	0.0181 (6)	
C10	0.5304 (3)	0.7826 (2)	0.7442 (2)	0.0258 (7)	
C11	0.8490 (3)	0.55266 (18)	0.6861 (2)	0.0209 (7)	

### Atomic displacement parameters (Å^2^)

**Table:**

	*U*^11^	*U*^22^	*U*^33^	*U*^12^	*U*^13^	*U*^23^
Yb1	0.01250 (6)	0.01161 (6)	0.01467 (7)	0.00037 (4)	0.00311 (5)	0.00005 (4)
Yb2	0.01271 (7)	0.01391 (7)	0.01816 (7)	−0.00026 (4)	0.00445 (5)	−0.00032 (5)
Yb3	0.01161 (7)	0.01416 (7)	0.01668 (7)	−0.00050 (4)	0.00478 (5)	−0.00059 (5)
F1	0.0149 (8)	0.0153 (8)	0.0143 (8)	−0.0003 (7)	0.0037 (7)	0.0003 (7)
F2	0.0134 (8)	0.0141 (8)	0.0177 (9)	0.0005 (7)	0.0039 (7)	0.0011 (7)
F3	0.0131 (8)	0.0143 (8)	0.0161 (9)	0.0005 (6)	0.0039 (7)	0.0006 (6)
F4	0.0144 (8)	0.0140 (8)	0.0148 (8)	−0.0004 (7)	0.0027 (7)	0.0005 (7)
F5	0.100 (2)	0.0670 (19)	0.0231 (13)	−0.0320 (17)	0.0179 (14)	0.0085 (12)
F6	0.0210 (11)	0.0392 (14)	0.081 (2)	−0.0076 (10)	0.0033 (12)	0.0276 (13)
F7	0.0431 (14)	0.0205 (11)	0.0783 (19)	−0.0005 (10)	0.0238 (14)	0.0125 (11)
F8	0.051 (3)	0.057 (3)	0.056 (4)	0.001 (2)	0.034 (3)	0.027 (3)
F9	0.039 (2)	0.023 (2)	0.027 (2)	0.0093 (17)	0.005 (2)	0.0089 (15)
F10	0.072 (4)	0.035 (2)	0.016 (2)	0.005 (2)	−0.006 (2)	0.0019 (16)
F8A	0.051 (8)	0.061 (9)	0.022 (8)	0.014 (7)	−0.013 (6)	0.004 (6)
F9A	0.072 (8)	0.067 (9)	0.037 (7)	0.005 (6)	0.031 (6)	0.022 (6)
F10A	0.094 (15)	0.027 (7)	0.040 (8)	0.009 (8)	0.017 (11)	0.016 (5)
F11	0.046 (3)	0.134 (5)	0.060 (3)	−0.020 (3)	0.021 (3)	0.031 (4)
F12	0.132 (7)	0.055 (4)	0.065 (5)	−0.014 (4)	0.069 (5)	−0.016 (3)
F13	0.089 (4)	0.062 (3)	0.070 (4)	0.027 (3)	0.042 (3)	0.045 (3)
O8	0.018 (4)	0.029 (3)	0.014 (3)	−0.002 (3)	0.003 (3)	−0.002 (2)
O9	0.016 (2)	0.021 (4)	0.017 (2)	0.003 (2)	0.0043 (15)	0.000 (2)
C5	0.022 (2)	0.0148 (19)	0.018 (2)	0.0031 (16)	0.0091 (17)	0.0011 (15)
C6	0.023 (2)	0.039 (3)	0.029 (3)	0.003 (2)	0.014 (2)	0.004 (2)
F11A	0.061 (7)	0.059 (7)	0.040 (6)	−0.001 (5)	0.027 (5)	−0.012 (5)
F12A	0.119 (12)	0.049 (7)	0.097 (10)	0.000 (7)	0.073 (10)	0.022 (7)
F13A	0.097 (10)	0.098 (11)	0.065 (9)	−0.032 (8)	0.046 (8)	0.009 (8)
O8A	0.018 (4)	0.029 (3)	0.014 (3)	−0.002 (3)	0.003 (3)	−0.002 (2)
O9A	0.025 (5)	0.035 (6)	0.033 (6)	−0.013 (5)	0.005 (5)	−0.006 (5)
C5A	0.017 (6)	0.024 (7)	0.012 (6)	−0.002 (5)	0.004 (5)	0.005 (5)
C6A	0.047 (8)	0.044 (7)	0.016 (6)	0.000 (7)	0.008 (6)	0.001 (6)
C6B	0.023 (2)	0.039 (3)	0.029 (3)	0.003 (2)	0.014 (2)	0.004 (2)
F11B	0.039 (7)	0.107 (12)	0.029 (7)	−0.004 (8)	0.005 (5)	0.044 (8)
F12B	0.048 (5)	0.057 (5)	0.045 (5)	0.021 (4)	0.034 (4)	0.009 (4)
F13B	0.068 (6)	0.040 (5)	0.037 (5)	−0.020 (4)	0.025 (4)	0.004 (4)
F14	0.0543 (16)	0.0700 (19)	0.0323 (13)	0.0223 (14)	0.0084 (12)	−0.0208 (13)
F15	0.0373 (13)	0.0237 (11)	0.0648 (17)	0.0139 (9)	0.0218 (12)	0.0038 (11)
F16	0.0192 (11)	0.0327 (12)	0.0807 (19)	0.0015 (9)	0.0185 (11)	−0.0160 (12)
F17	0.037 (2)	0.064 (4)	0.071 (4)	0.017 (2)	0.006 (2)	−0.039 (4)
F18	0.066 (4)	0.028 (2)	0.072 (4)	−0.019 (3)	0.028 (3)	−0.024 (2)
F19	0.123 (7)	0.039 (3)	0.023 (2)	0.019 (3)	−0.004 (3)	−0.0125 (19)
F17A	0.035 (4)	0.044 (4)	0.047 (5)	0.005 (3)	−0.013 (3)	−0.031 (4)
F18A	0.063 (6)	0.063 (5)	0.068 (6)	−0.012 (4)	0.049 (5)	−0.032 (5)
F19A	0.101 (10)	0.022 (3)	0.026 (3)	0.015 (4)	0.010 (4)	−0.004 (2)
C12	0.0169 (18)	0.034 (3)	0.023 (2)	0.0023 (17)	0.0010 (15)	−0.003 (2)
F20	0.0298 (19)	0.077 (3)	0.053 (3)	−0.001 (2)	−0.0149 (18)	0.029 (2)
F21	0.0161 (17)	0.094 (4)	0.037 (2)	−0.005 (3)	0.0057 (16)	−0.024 (3)
F22	0.0268 (18)	0.058 (3)	0.063 (3)	−0.0015 (18)	−0.0120 (18)	−0.037 (2)
C12A	0.0169 (18)	0.034 (3)	0.023 (2)	0.0023 (17)	0.0010 (15)	−0.003 (2)
F20A	0.030 (7)	0.070 (8)	0.056 (7)	−0.029 (7)	−0.001 (5)	0.010 (7)
F21A	0.031 (6)	0.138 (13)	0.029 (6)	0.001 (8)	0.007 (5)	−0.030 (8)
F22A	0.034 (6)	0.045 (6)	0.089 (9)	0.014 (5)	−0.019 (6)	0.008 (6)
N1	0.035 (2)	0.014 (4)	0.033 (3)	0.002 (2)	0.008 (2)	−0.002 (2)
O1	0.035 (2)	0.014 (4)	0.033 (3)	0.002 (2)	0.008 (2)	−0.002 (2)
O2	0.061 (4)	0.082 (4)	0.050 (3)	−0.013 (4)	−0.013 (3)	0.018 (3)
O3	0.057 (3)	0.067 (4)	0.067 (5)	0.002 (3)	−0.003 (4)	0.008 (4)
O2A	0.061 (4)	0.082 (4)	0.050 (3)	−0.013 (4)	−0.013 (3)	0.018 (3)
O3A	0.057 (3)	0.067 (4)	0.067 (5)	0.002 (3)	−0.003 (4)	0.008 (4)
O4	0.0186 (11)	0.0205 (12)	0.0256 (12)	−0.0030 (9)	0.0070 (10)	0.0004 (9)
O5	0.0182 (11)	0.0177 (11)	0.0229 (12)	−0.0036 (9)	0.0060 (9)	−0.0008 (9)
O6	0.0212 (11)	0.0203 (11)	0.0174 (11)	0.0005 (9)	0.0029 (9)	0.0028 (9)
O7	0.0213 (11)	0.0220 (12)	0.0168 (11)	0.0013 (9)	0.0026 (9)	0.0021 (9)
O10	0.0173 (11)	0.0177 (11)	0.0199 (11)	0.0038 (9)	0.0042 (9)	0.0008 (9)
O11	0.0180 (11)	0.0182 (11)	0.0300 (13)	0.0015 (9)	0.0097 (10)	−0.0013 (9)
O12	0.0192 (11)	0.0175 (11)	0.0184 (11)	−0.0010 (9)	0.0033 (9)	−0.0021 (9)
O13	0.0231 (12)	0.0208 (12)	0.0178 (11)	−0.0013 (9)	0.0031 (9)	−0.0024 (9)
O14	0.0181 (11)	0.0262 (12)	0.0227 (12)	−0.0018 (9)	0.0030 (9)	−0.0039 (10)
O15	0.0163 (11)	0.0228 (12)	0.0206 (12)	−0.0024 (9)	0.0010 (9)	−0.0029 (9)
O16	0.0165 (11)	0.0257 (12)	0.0236 (13)	−0.0030 (10)	0.0043 (10)	−0.0022 (10)
O17	0.0266 (18)	0.0310 (18)	0.0194 (17)	−0.0003 (15)	0.0009 (15)	−0.0031 (14)
O17A	0.016 (2)	0.021 (4)	0.017 (2)	0.003 (2)	0.0043 (15)	0.000 (2)
C1	0.0202 (15)	0.0158 (14)	0.0131 (14)	−0.0061 (12)	0.0013 (12)	−0.0008 (11)
C2	0.0206 (16)	0.0244 (17)	0.0257 (18)	−0.0033 (13)	0.0046 (14)	0.0070 (13)
C3	0.0121 (14)	0.0215 (16)	0.0171 (15)	0.0013 (12)	0.0031 (12)	0.0047 (12)
C4	0.037 (2)	0.0267 (18)	0.0210 (17)	0.0025 (15)	0.0070 (15)	0.0056 (14)
C7	0.0156 (15)	0.0197 (15)	0.0167 (15)	0.0035 (12)	0.0020 (12)	−0.0027 (12)
C8	0.0190 (16)	0.0241 (17)	0.0289 (18)	0.0024 (13)	0.0048 (14)	−0.0065 (14)
C9	0.0154 (14)	0.0196 (15)	0.0196 (16)	−0.0035 (12)	0.0045 (12)	−0.0036 (12)
C10	0.0313 (19)	0.0241 (17)	0.0215 (17)	0.0025 (14)	0.0046 (14)	−0.0034 (13)
C11	0.0179 (16)	0.0179 (15)	0.0236 (17)	−0.0011 (12)	−0.0026 (13)	−0.0017 (13)

### Geometric parameters (Å, º)

**Table:**

Yb1—F1	2.2375 (17)	F14—C8	1.317 (4)
Yb1—F2	2.2382 (17)	F15—C8	1.327 (4)
Yb1—F3	2.2431 (17)	F16—C8	1.321 (4)
Yb1—F4^i^	2.2444 (17)	F17—C10	1.305 (5)
Yb1—O5	2.273 (2)	F18—C10	1.342 (5)
Yb1—O10	2.291 (2)	F19—C10	1.305 (6)
Yb1—O6	2.306 (2)	F17A—C10	1.316 (7)
Yb1—O12	2.309 (2)	F18A—C10	1.313 (7)
Yb2—O8A	2.23 (4)	F19A—C10	1.297 (7)
Yb2—O15^i^	2.286 (2)	C12—F21	1.315 (7)
Yb2—F2^i^	2.2895 (17)	C12—F22	1.316 (7)
Yb2—O8	2.299 (13)	C12—F20	1.326 (8)
Yb2—O7	2.303 (2)	C12—C11	1.549 (6)
Yb2—F4	2.3035 (17)	C12A—F20A	1.296 (18)
Yb2—F3	2.3061 (17)	C12A—F22A	1.301 (18)
Yb2—F1	2.3276 (17)	C12A—F21A	1.302 (18)
Yb2—O13^i^	2.329 (2)	C12A—C11	1.531 (15)
Yb2—O17	2.544 (3)	N1—H12	0.9100
Yb2—C5A	3.054 (11)	N1—H13	0.9100
Yb3—O17A	2.27 (4)	N1—H14	0.9099
Yb3—O14	2.290 (2)	N1—H15	0.9099
Yb3—O9	2.312 (11)	O1—H1	0.8400
Yb3—O11^i^	2.321 (2)	O1—H2	0.8401
Yb3—F3^i^	2.3210 (17)	O1—H3	0.8401
Yb3—O4	2.323 (2)	O2—H4	0.830 (2)
Yb3—F2	2.3316 (17)	O2—H5	0.830 (2)
Yb3—F1	2.3321 (17)	O3—H6	0.852 (17)
Yb3—F4	2.3509 (17)	O3—H7	0.849 (18)
Yb3—O16	2.448 (2)	O2A—H4A	0.830 (2)
F5—C2	1.306 (4)	O2A—H5A	0.830 (3)
F6—C2	1.312 (4)	O3A—H6A	0.83 (2)
F7—C2	1.327 (4)	O3A—H7A	0.84 (2)
F8—C4	1.322 (6)	O4—C1	1.244 (4)
F9—C4	1.341 (6)	O5—C1	1.246 (4)
F10—C4	1.333 (6)	O6—C3	1.244 (4)
F8A—C4	1.315 (14)	O7—C3	1.239 (4)
F9A—C4	1.302 (14)	O10—C7	1.248 (4)
F10A—C4	1.311 (15)	O11—C7	1.236 (4)
F11—C6	1.317 (2)	O12—C9	1.246 (4)
F12—C6	1.319 (2)	O13—C9	1.239 (4)
F13—C6	1.315 (2)	O14—C11	1.231 (4)
O8—C5	1.240 (10)	O15—C11	1.251 (4)
O9—C5	1.250 (11)	O16—H8	0.831 (19)
C5—C6	1.519 (6)	O16—H8A	0.84 (2)
F11A—C6A	1.320 (2)	O16—H9	0.817 (19)
F12A—C6A	1.320 (2)	O17—H10	0.81 (2)
F13A—C6A	1.320 (2)	O17—H11	0.84 (2)
O8A—C5A	1.26 (4)	O17A—H10A	0.830 (2)
O9A—C5A	1.207 (17)	O17A—H11A	0.830 (2)
C5A—C6A	1.532 (14)	C1—C2	1.544 (4)
C6B—F11B	1.266 (14)	C3—C4	1.545 (5)
C6B—F12B	1.318 (11)	C7—C8	1.544 (4)
C6B—F13B	1.355 (12)	C9—C10	1.546 (4)
			
F1—Yb1—F2	68.51 (6)	Yb1^i^—F4—Yb2	111.42 (7)
F1—Yb1—F3	68.68 (6)	Yb1^i^—F4—Yb3	112.27 (7)
F2—Yb1—F3	105.52 (6)	Yb2—F4—Yb3	115.81 (7)
F1—Yb1—F4^i^	105.62 (6)	C5—O8—Yb2	135.7 (7)
F2—Yb1—F4^i^	68.79 (6)	C5—O9—Yb3	136.4 (7)
F3—Yb1—F4^i^	68.20 (6)	O8—C5—O9	129.5 (8)
F1—Yb1—O5	79.54 (7)	O8—C5—C6	114.2 (6)
F2—Yb1—O5	79.72 (7)	O9—C5—C6	116.3 (6)
F3—Yb1—O5	142.37 (7)	F13—C6—F11	107.9 (5)
F4^i^—Yb1—O5	142.72 (7)	F13—C6—F12	107.5 (6)
F1—Yb1—O10	142.97 (7)	F11—C6—F12	106.4 (6)
F2—Yb1—O10	141.71 (7)	F13—C6—C5	110.7 (4)
F3—Yb1—O10	79.93 (7)	F11—C6—C5	114.2 (4)
F4^i^—Yb1—O10	79.02 (7)	F12—C6—C5	109.9 (6)
O5—Yb1—O10	119.28 (8)	C5A—O8A—Yb2	120 (2)
F1—Yb1—O6	79.31 (7)	O9A—C5A—O8A	126 (2)
F2—Yb1—O6	142.28 (7)	O9A—C5A—C6A	120.0 (12)
F3—Yb1—O6	79.24 (7)	O8A—C5A—C6A	114 (2)
F4^i^—Yb1—O6	141.72 (7)	O9A—C5A—Yb2	88.0 (8)
O5—Yb1—O6	75.44 (8)	C6A—C5A—Yb2	151.3 (8)
O10—Yb1—O6	75.89 (8)	F13A—C6A—F12A	106.3 (11)
F1—Yb1—O12	142.51 (7)	F13A—C6A—F11A	105.6 (11)
F2—Yb1—O12	79.38 (7)	F12A—C6A—F11A	105.7 (9)
F3—Yb1—O12	141.46 (7)	F13A—C6A—C5A	117.8 (13)
F4^i^—Yb1—O12	78.91 (7)	F12A—C6A—C5A	109.5 (10)
O5—Yb1—O12	76.05 (8)	F11A—C6A—C5A	111.1 (10)
O10—Yb1—O12	74.46 (8)	F11B—C6B—F12B	108.8 (9)
O6—Yb1—O12	120.44 (8)	F11B—C6B—F13B	105.8 (8)
O8A—Yb2—O15^i^	122.8 (7)	F12B—C6B—F13B	101.0 (8)
O15^i^—Yb2—F2^i^	77.47 (7)	F21—C12—F22	107.6 (6)
O15^i^—Yb2—O8	130.11 (18)	F21—C12—F20	108.1 (5)
F2^i^—Yb2—O8	141.6 (3)	F22—C12—F20	105.7 (5)
O8A—Yb2—O7	78.4 (11)	F21—C12—C11	112.7 (5)
O15^i^—Yb2—O7	81.21 (8)	F22—C12—C11	111.6 (5)
F2^i^—Yb2—O7	137.25 (7)	F20—C12—C11	110.9 (5)
O8—Yb2—O7	79.2 (3)	F20A—C12A—F22A	106.1 (14)
O8A—Yb2—F4	82.4 (9)	F20A—C12A—F21A	107.0 (15)
O15^i^—Yb2—F4	140.92 (7)	F22A—C12A—F21A	107.2 (15)
F2^i^—Yb2—F4	66.91 (6)	F20A—C12A—C11	113.2 (16)
O8—Yb2—F4	77.2 (3)	F22A—C12A—C11	112.6 (13)
O7—Yb2—F4	136.54 (7)	F21A—C12A—C11	110.4 (14)
O8A—Yb2—F3	142.9 (10)	H12—N1—H13	109.5
O15^i^—Yb2—F3	78.19 (7)	H12—N1—H14	109.5
F2^i^—Yb2—F3	64.51 (6)	H13—N1—H14	109.5
O8—Yb2—F3	137.7 (3)	H12—N1—H15	109.5
O7—Yb2—F3	75.12 (7)	H13—N1—H15	109.5
F4—Yb2—F3	99.50 (6)	H14—N1—H15	109.5
O8A—Yb2—F1	82.3 (8)	H1—O1—H2	111.7
O15^i^—Yb2—F1	140.92 (7)	H1—O1—H3	111.7
F2^i^—Yb2—F1	99.78 (6)	H2—O1—H3	111.6
O8—Yb2—F1	75.12 (19)	H4—O2—H5	105.4 (6)
O7—Yb2—F1	74.92 (7)	H6—O3—H7	107 (5)
F4—Yb2—F1	64.05 (6)	H4A—O2A—H5A	105.4 (6)
F3—Yb2—F1	66.11 (6)	H6A—O3A—H7A	106 (5)
O15^i^—Yb2—O13^i^	81.76 (8)	C1—O4—Yb3	138.0 (2)
F2^i^—Yb2—O13^i^	76.31 (7)	C1—O5—Yb1	131.0 (2)
O8—Yb2—O13^i^	81.8 (3)	C3—O6—Yb1	129.9 (2)
O7—Yb2—O13^i^	136.28 (8)	C3—O7—Yb2	138.1 (2)
F4—Yb2—O13^i^	74.85 (7)	C7—O10—Yb1	129.8 (2)
F3—Yb2—O13^i^	138.88 (7)	C7—O11—Yb3^i^	138.6 (2)
F1—Yb2—O13^i^	136.15 (7)	C9—O12—Yb1	130.9 (2)
O15^i^—Yb2—O17	65.63 (10)	C9—O13—Yb2^i^	136.9 (2)
F2^i^—Yb2—O17	131.62 (10)	C11—O14—Yb3	138.5 (2)
O8—Yb2—O17	64.50 (19)	C11—O15—Yb2^i^	133.8 (2)
O7—Yb2—O17	67.64 (10)	Yb3—O16—H8	109 (4)
F4—Yb2—O17	129.63 (9)	Yb3—O16—H8A	118 (10)
F3—Yb2—O17	130.81 (9)	Yb3—O16—H9	111 (3)
F1—Yb2—O17	128.60 (9)	H8—O16—H9	105 (4)
O13^i^—Yb2—O17	68.65 (10)	H8A—O16—H9	104 (5)
O8A—Yb2—C5A	20.9 (8)	Yb2—O17—H10	94 (4)
O15^i^—Yb2—C5A	101.9 (2)	Yb2—O17—H11	108 (6)
F2^i^—Yb2—C5A	149.4 (2)	H10—O17—H11	109 (4)
O7—Yb2—C5A	71.3 (2)	Yb3—O17A—H10A	92 (5)
F4—Yb2—C5A	100.8 (2)	Yb3—O17A—H11A	116 (10)
F3—Yb2—C5A	145.9 (2)	H10A—O17A—H11A	106 (5)
F1—Yb2—C5A	99.1 (2)	O4—C1—O5	129.6 (3)
O13^i^—Yb2—C5A	73.4 (2)	O4—C1—C2	115.3 (3)
O17A—Yb3—O14	133.8 (9)	O5—C1—C2	115.0 (3)
O14—Yb3—O9	135.5 (3)	F5—C2—F6	109.2 (3)
O14—Yb3—O11^i^	82.90 (8)	F5—C2—F7	106.5 (3)
O9—Yb3—O11^i^	84.38 (16)	F6—C2—F7	106.3 (3)
O14—Yb3—F3^i^	74.74 (7)	F5—C2—C1	109.5 (3)
O9—Yb3—F3^i^	141.5 (2)	F6—C2—C1	112.9 (3)
O11^i^—Yb3—F3^i^	75.69 (7)	F7—C2—C1	112.1 (3)
O17A—Yb3—O4	85.1 (7)	O7—C3—O6	130.2 (3)
O14—Yb3—O4	84.13 (9)	O7—C3—C4	114.6 (3)
O9—Yb3—O4	78.74 (19)	O6—C3—C4	115.1 (3)
O11^i^—Yb3—O4	139.80 (8)	F9A—C4—F10A	108.5 (11)
F3^i^—Yb3—O4	136.04 (7)	F9A—C4—F8A	106.7 (9)
O17A—Yb3—F2	141.7 (7)	F10A—C4—F8A	107.9 (11)
O14—Yb3—F2	76.44 (7)	F8—C4—F10	108.0 (4)
O9—Yb3—F2	135.08 (17)	F8—C4—F9	107.7 (4)
O11^i^—Yb3—F2	137.83 (7)	F10—C4—F9	106.6 (5)
F3^i^—Yb3—F2	63.63 (6)	F9A—C4—C3	111.9 (9)
O4—Yb3—F2	74.24 (7)	F10A—C4—C3	114.5 (12)
O17A—Yb3—F1	78.4 (8)	F8A—C4—C3	107.0 (10)
O14—Yb3—F1	140.07 (7)	F8—C4—C3	109.0 (4)
O9—Yb3—F1	73.3 (2)	F10—C4—C3	113.1 (4)
O11^i^—Yb3—F1	133.95 (7)	F9—C4—C3	112.2 (4)
F3^i^—Yb3—F1	97.34 (6)	O11—C7—O10	130.0 (3)
O4—Yb3—F1	74.91 (7)	O11—C7—C8	115.3 (3)
F2—Yb3—F1	65.39 (6)	O10—C7—C8	114.6 (3)
O17A—Yb3—F4	74.9 (9)	F14—C8—F16	107.6 (3)
O14—Yb3—F4	136.97 (7)	F14—C8—F15	107.5 (3)
O9—Yb3—F4	77.7 (2)	F16—C8—F15	107.1 (3)
O11^i^—Yb3—F4	72.99 (7)	F14—C8—C7	109.4 (3)
F3^i^—Yb3—F4	65.16 (6)	F16—C8—C7	112.3 (3)
O4—Yb3—F4	136.25 (7)	F15—C8—C7	112.8 (3)
F2—Yb3—F4	98.13 (6)	O13—C9—O12	129.9 (3)
F1—Yb3—F4	63.25 (6)	O13—C9—C10	115.8 (3)
O17A—Yb3—O16	65.8 (9)	O12—C9—C10	114.3 (3)
O14—Yb3—O16	68.32 (8)	F19—C10—F17	108.5 (5)
O9—Yb3—O16	67.3 (3)	F19A—C10—F18A	108.5 (6)
O11^i^—Yb3—O16	69.60 (8)	F19A—C10—F17A	107.1 (6)
F3^i^—Yb3—O16	131.44 (7)	F18A—C10—F17A	106.6 (6)
O4—Yb3—O16	70.24 (8)	F19—C10—F18	105.9 (5)
F2—Yb3—O16	131.62 (7)	F17—C10—F18	105.6 (4)
F1—Yb3—O16	131.20 (7)	F19A—C10—C9	114.2 (4)
F4—Yb3—O16	130.25 (7)	F19—C10—C9	114.2 (3)
Yb1—F1—Yb2	111.91 (7)	F17—C10—C9	112.0 (3)
Yb1—F1—Yb3	112.59 (7)	F18A—C10—C9	109.5 (4)
Yb2—F1—Yb3	115.60 (7)	F17A—C10—C9	110.7 (4)
Yb1—F2—Yb2^i^	112.17 (7)	F18—C10—C9	110.2 (3)
Yb1—F2—Yb3	112.58 (7)	O14—C11—O15	129.8 (3)
Yb2^i^—F2—Yb3	115.21 (7)	O14—C11—C12A	110.5 (8)
Yb1—F3—Yb2	112.51 (7)	O15—C11—C12A	119.4 (8)
Yb1—F3—Yb3^i^	113.46 (7)	O14—C11—C12	117.3 (4)
Yb2—F3—Yb3^i^	114.98 (7)	O15—C11—C12	112.9 (4)

Symmetry code: (i) −*x*+1, −*y*+1, −*z*+1.

### Hydrogen-bond geometry (Å, º)

**Table:**

*D*—H···*A*	*D*—H	H···*A*	*D*···*A*	*D*—H···*A*
N1—H12···O6	0.91	2.21	2.883 (8)	131
N1—H15···O17^ii^	0.91	1.86	2.766 (7)	172
N1—H13···O10	0.91	2.08	2.853 (10)	142
N1—H14···O2	0.91	1.95	2.837 (9)	165
O1—H2···O2*A*	0.84	1.78	2.619 (18)	175
O1—H3···O9*A*^ii^	0.84	2.01	2.842 (16)	173
O1—H1···O10	0.84	2.30	3.03 (3)	145
O2—H4···O3	0.83 (1)	1.91 (3)	2.704 (11)	159 (7)
O2—H5···O12	0.83 (1)	2.31 (5)	2.978 (7)	138 (6)
O3—H6···O2^iii^	0.85 (2)	2.22 (2)	3.055 (10)	167 (8)
O3—H7···O17^iv^	0.85 (2)	2.01 (2)	2.835 (9)	163 (8)
O2*A*—H4*A*···F19*A*	0.83 (1)	1.90 (7)	2.68 (2)	156 (17)
O2*A*—H5*A*···O3*A*^iii^	0.83 (1)	2.35 (14)	2.96 (3)	131 (16)
O3*A*—H7*A*···O2*A*	0.84 (2)	2.20 (15)	2.91 (4)	143 (23)
O3*A*—H6*A*···O9*A*^iv^	0.83 (2)	2.6 (2)	3.03 (4)	115 (19)
O16—H8···O9	0.83 (2)	2.28 (6)	2.639 (10)	107 (4)
O16—H8*A*···O16^v^	0.84 (2)	2.07 (2)	2.903 (5)	179 (17)
O16—H9···F16^vi^	0.82 (2)	2.17 (2)	2.954 (3)	160 (4)
O17—H11···O8	0.84 (2)	2.25 (8)	2.593 (7)	105 (7)
O17—H10···O13^i^	0.81 (2)	2.22 (5)	2.754 (4)	123 (5)
O17*A*—H10*A*···O16	0.83 (1)	1.92 (2)	2.56 (3)	134 (5)

Symmetry codes: (i) −*x*+1, −*y*+1, −*z*+1; (ii) −*x*+1/2, *y*+1/2, −*z*+1/2; (iii) −*x*+1, −*y*+2, −*z*+1; (iv) *x*+1/2, −*y*+3/2, *z*+1/2; (v) −*x*+2, −*y*+1, −*z*+1; (vi) *x*+1, *y*, *z*.
